# Novel Conserved Genotypes Correspond to Antibiotic Resistance Phenotypes of *E. coli* Clinical Isolates

**DOI:** 10.1371/journal.pone.0065961

**Published:** 2013-06-18

**Authors:** Michelle C. Swick, Michael A. Evangelista, Truston J. Bodine, Jeremy R. Easton-Marks, Patrick Barth, Minita J. Shah, Christina A. Bormann Chung, Sarah Stanley, Stephen F. McLaughlin, Clarence C. Lee, Vrunda Sheth, Quynh Doan, Richard J. Hamill, David Steffen, Lauren B. Becnel, Richard Sucgang, Lynn Zechiedrich

**Affiliations:** 1 Interdepartmental Program in Cell and Molecular Biology, Baylor College of Medicine, Houston, Texas, United States of America; 2 Department of Molecular Virology and Microbiology, Baylor College of Medicine, Houston, Texas, United States of America; 3 Verna and Marrs McLean Department of Biochemistry and Molecular Biology, Houston, Texas, United States of America; 4 Interdepartmental Program in Translational Biology and Molecular Medicine, Baylor College of Medicine, Houston, Texas, United States of America; 5 Dan L. Duncan Cancer Center, Baylor College of Medicine, Houston, Texas, United States of America; 6 Biomedical Informatics Group, Baylor College of Medicine, Houston, Texas, United States of America; 7 Department of Pharmacology, Baylor College of Medicine, Houston, Texas, United States of America; 8 Life Technologies, Beverly, Massachusetts, United States of America; 9 Life Technologies, Foster City, California, United States of America; 10 Department of Medicine, Baylor College of Medicine, Houston, Texas, United States of America; 11 Department of Molecular and Cellular Biology, Baylor College of Medicine, Houston, Texas, United States of America; University Medical Center Utrecht, The Netherlands

## Abstract

Current efforts to understand antibiotic resistance on the whole genome scale tend to focus on known genes even as high throughput sequencing strategies uncover novel mechanisms. To identify genomic variations associated with antibiotic resistance, we employed a modified genome-wide association study; we sequenced genomic DNA from pools of *E. coli* clinical isolates with similar antibiotic resistance phenotypes using SOLiD technology to uncover single nucleotide polymorphisms (SNPs) unanimously conserved in each pool. The multidrug-resistant pools were genotypically similar to SMS-3-5, a previously sequenced multidrug-resistant isolate from a polluted environment. The similarity was evenly spread across the entire genome and not limited to plasmid or pathogenicity island loci. Among the pools of clinical isolates, genomic variation was concentrated adjacent to previously reported inversion and duplication differences between the SMS-3-5 isolate and the drug-susceptible laboratory strain, DH10B. SNPs that result in non-synonymous changes in *gyrA* (encoding the well-known S83L allele associated with fluoroquinolone resistance), *mutM*, *ligB*, and *recG* were unanimously conserved in every fluoroquinolone-resistant pool. Alleles of the latter three genes are tightly linked among most sequenced *E. coli* genomes, and had not been implicated in antibiotic resistance previously. The changes in these genes map to amino acid positions in alpha helices that are involved in DNA binding. Plasmid-encoded complementation of null strains with either allelic variant of *mutM* or *ligB* resulted in variable responses to ultraviolet light or hydrogen peroxide treatment as markers of induced DNA damage, indicating their importance in DNA metabolism and revealing a potential mechanism for fluoroquinolone resistance. Our approach uncovered evidence that additional DNA binding enzymes may contribute to fluoroquinolone resistance and further implicate environmental bacteria as a reservoir for antibiotic resistance.

## Introduction

Antibiotic-resistant bacterial pathogens present a grave threat to human health. Each year, approximately two million people in the United States develop bacterial infections while in the hospital [1], and more than half of these infections involve bacteria that are multidrug-resistant [1], [2]. In some cases, gram-negative bacteria are resistant to nearly every existing antibiotic [2]. These hospital-acquired infections resulted in nearly 100,000 deaths in 2002 [3], [4], and are predicted to cost the U.S. between $5 and $10 billion dollars annually [5]. Multidrug-resistant bacteria contribute to increased mortality rates and lengthier hospital stays [1]. Furthermore, the cost to treat multidrug-resistant infections is ∼30% more than drug-susceptible infections [1].

Pathogens evolve antibiotic resistance when exposed to empirically prescribed antibiotics. Resistance mechanisms vary for each antibiotic class, and may evolve during the course of antibiotic exposure or be acquired through horizontal gene transfer. The accumulation of genetic alterations can result in a complex, polygenic phenotype (reviewed in [6]). For example, resistance to the bactericidal fluoroquinolone class of antibiotics, a popular empirical prescription choice, develops through multiple and temporally coordinated mutations and mechanisms [7]–[10]. Mutations in chromosomally encoded genes are the primary mechanism of resistance to fluoroquinolones. In *E. coli*, these mutations include the clinically relevant L83 variant of *gyrA* (one of two genes encoding gyrase) and I80 variant of *parC* (one of two genes encoding topoisomerase IV) [7], [8], [11]–[13]. Pathogens may also acquire plasmid-encoded resistance genes such as the *qnr* variants and *aac-(6′)-Ib*-cr ([14]–[17], and reviewed in [18]). Additionally, changes in efflux pump expression can contribute to fluoroquinolone and multidrug resistance [9], [19]–[21]. Many reports track these types of fluoroquinolone resistance mechanisms in gram-negative isolates. However, the focus on analyzing known mechanisms can occlude the discovery of novel resistance mechanisms. Some fluoroquinolone-resistant clinical isolates have fluoroquinolone minimum inhibitory concentrations (MICs) that cannot be explained by known mechanisms [20], suggesting that additional mechanisms may be present.

High throughput sequencing methods have proven exceptionally useful in identifying novel single nucleotide polymorphisms (SNPs) linked to important phenotypes. Such genome-wide association studies (GWAS) are frequently employed to study genomic alterations associated with human disease [22]. Researchers have used comparative genomics to study the resistome [23], [24] and have had success with clonal species like *Streptococcus* [25]. Although whole genome sequencing technology has become dramatically less expensive, the background of genomic variation present in large datasets presents a major computational challenge. Furthermore, given so much sequence information, the focus is still primarily on analysis of variants in genes previously implicated in resistance. We hypothesized that a pool of bacterial isolates that share an antibiotic resistance phenotype should also share a genomic signature. Sequencing genomic DNA from a pool self-normalizes, and thus dampens non-specific variations to reveal genetic variants relevant to the shared phenotype. Sequencing pools of isolates would then leverage the power of GWAS at a much lower cost.

Here, we adapted a GWAS for use with pooled genomic samples. We present evidence that the antibiotic resistance phenotype correlated with the genotype of the pool. We show that pools of multidrug-resistant *E. coli* clinical isolates with high fluoroquinolone MICs resemble the environmental isolate, SMS-3-5. Our data provide support for the hypothesis that environmental bacteria may serve as a reservoir for antibiotic resistance [26], [27]. This study uncovered three tightly linked SNPs that generate non-synonymous variants of genes encoding DNA binding proteins, which are highly conserved in fluoroquinolone-resistant *E. coli*.

## Results

### Experimental Design and Sequencing Strategy

From our curated collection of >4,000 clinical isolates [20], [28], we selected 164 non-clonal *E. coli*, each one from a different patient, which were representative of all of the antibiotic resistance phenotypes existing in the collection. Table S1 and Table S2 show the antibiotics tested and the patient demographics for these isolates, respectively. The isolates ranged from susceptible to all tested antibiotics to multidrug-resistant and had fluoroquinolone MICs spanning six orders of magnitude [20].

We grouped the isolates into 16 pools by *k*-means clustering using Cluster 3.0 [29]. We reasoned that to best identify genetic alterations associated with antibiotic resistance, we would cluster isolates only by antibiotic resistance phenotypes and, thus, we did not include the variables of patient metadata or serotype. The parameters for the clustering algorithm included the MIC values for four fluoroquinolones and the drug susceptibility status for 17 additional antibiotics (Table S1). The number of isolates in each pool ranged from two to thirty-two. Table 1 describes the pools and their profile of drug resistance.

**Table 1:** Antibiotic resistance phenotypes of pools.

Pool	n	MDRstatus	FQ MIC (µg/mL) [20]				Consensus resistance phenotype
			CIP	GAT	LVX	NOR	
S01	9	No	0.01	0.01–0.02	0.02–0.07	0.03–0.09	AMP, SXT
S02	9	No	0.01–0.09	0.01–0.09	0.03–0.17	0.04–0.34	None
M01	10	MDR_≥3_	30–100	10–30	20–50	200–700	FQs, AMP, FOX
M02	10	MDR_≥5_	100–500	10–30	20–50	200–700	FQs, AMP, FOX, CFZ, GEN, SXT, TIM
M03	13	No	10–30	8–30	8–30	50–200	FQs
M04	23	No	100–400	30–100	50–100	200–700	FQs, AMP
M05	16	MDR_≥5_	20–50	10–20	30–50	50–300	FQs, AMP, FOX, SXT, TIM
M06	13	MDR_≥3_	50–200	10–20	30–100	200–400	FQs, AMP, GEN, SXT
M07	33	MDR_≥3_	10–30	10	10–20	50–200	FQs, AMP, SXT, AMC
M08	3	No	50–200	100–300	50–200	100–200	FQs, SXT
M09	3	MDR_≥3_	20–30	20	10–20	100–200	FQs, AMP, GEN
M10	5	MDR_≥5_	100–400	20–100	30–100	50–500	FQs, AMP,SXT, TIM, NIT
M11	5	MDR_≥3_	10–100	10–50	20–50	50–200	FQs, AMP, SXT, NIT
H01	2	MDR_≥5_	100–300	10–30	10–200	1000	All tested except AMK and IPM
H02	5	MDR_≥3_	50–500	10–30	40–50	400–1000	FQs, AMP, FOX
H03	5	MDR_≥3_	200–500	50–200	100	400–1000	FQs, GEN, AMC, NIT

Individual isolates were pooled by *k*-means clustering and consensus resistance phenotypes (defined as the resistance profile of a majority of the isolates in a pool) were visually confirmed. Pools labeled “S” contain strains that are susceptible to fluoroquinolones and non-multidrug-resistant (non-MDR). Pools labeled “M” contain strains that are resistant to fluoroquinolones and may be MDR_≥3_ (resistant to at least 3 separate drug classes) or MDR_≥5_ (resistant to at least 5 separate drug classes) [21]. Pools labeled “H” contain strains that are MDR_≥3_ or MDR_≥5_ and have extremely high fluoroquinolone MICs. CIP = ciprofloxacin; GAT = gatifloxacin; LVX = levofloxacin; NOR = norfloxacin; AMP = ampicillin; SXT = trimethoprim-sulfamethoxazole; FQs = All four fluoroquinolones; FOX = cefoxitin; CFZ = cefazolin; GEN = gentamicin; TIM-ticarcillin-clavulanic acid; AMC = amoxicillin-clavulanic acid; NIT = nitrofurantoin; AMK = amikacin; IPM = imipenem.

Because of the multiple antibiotic resistance parameters used in the clustering algorithm, only a rough ordering of the pools can be easily described. Two pools of nine isolates each were fluoroquinolone-susceptible (“S”) and were also susceptible to most other antibiotics. Three pools (“H”) were made up of isolates with high fluoroquinolone MICs and were resistant to at least three separate drug classes (MDR_≥3_; [21]). The remaining pools (“M”) were intermediate between the other two sets, and were designated numerically (randomly) in the context of antibiotic resistance.

Besides decreasing cost, sequencing pools of isolates results in internal normalization, dampening non-specific sequence variation from individual strains while highlighting conserved genetic variants. We subjected pools of clinical isolate genomic DNA to next generation sequencing on the ABI SOLiD™ 3 Platform. Mate-pair reads were mapped using 3 separate genomes (detailed below) as scaffolding platforms, and SNPs were called as described in Figure S1. SNP calls were deposited in Dryad repository (http://dx.doi.org/10.5061/dryad.r8q71). The coverage of each SNP called averaged 150×. When SNPs were called as identical in nucleotide position and base identity for all reads in a pool, we refer to them as “unanimous.” Unanimous SNPs had *p*-values less than 1×10^−19^. When SNPs did not match these criteria, we refer to them as “mixed.” Mixed SNPs ranged in significance, as reflected by *p*-value, because of the lack of perfect agreement within a given pool. This initial analysis considers only unanimous SNPs.

### Antibiotic Resistance Phenotype Correlates with Genotype on a Whole-genome Scale

*E. coli* represents a diverse group of microorganisms with a predicted pangenome of over 16,000 homologous gene clusters. Only about 1,700 “core genes” are present in all 186 genomes sequenced to date [30]. Although next generation sequencing methods produce relatively short sequence reads, there is sufficient complexity such that calling differences from a reference genome sequence is a standard analysis technique. In most mathematical models, the sampled genome is presumably a descendant or at least closely related to the reference genome. The *E. coli* that made up the pools were isolated from clinics, and thus likely represent divergent evolutionary lines. Lacking a known common ancestral genome, we were confronted with the significant challenge of analyzing a potentially complex group of related genomic samples.

We chose to measure the genomic variation of the pooled samples against three published and annotated *E. coli* genomes that represented susceptible and multidrug-resistant phenotypes; over 90% of the sequence reads mapped to at least one of these reference genomes. This approach reduced the complexity of the problem, allowing detection of conserved sequences among the members of a pool that differed from each particular reference; it also enabled us to use published genomic annotations for gene identification. The *E. coli* genomes chosen for comparison varied in gene content and annotation but this variation had no bearing on the variant identification. The same short sequence reads were first mapped then annotated relative to each reference independently (Figure S1).

We selected genomes of two drug-susceptible strains and one multidrug-resistant strain. The laboratory K-12 strain DH10B [GenBank: CP000948.1] has been used for multiple genome projects [31] and provides a potential host for studying the effect of new SNPs in an isogenic strain. REL606 [GenBank: CP000819.1] derives from the B lineage [32], and is the ancestral strain of the long term evolution experiment of Lenski *et al.* ([33], and reviewed in [34]). We hypothesized that the genotypes from drug-susceptible pools would be more similar to these reference genomes than would the drug-resistant pools.

Mapped against the 4.6 Mb DH10B genome, we detected 1,135,007 unanimous and mixed SNPs across the 16 pools. Overall, the number of unanimous SNPs detected in each pool varied, but in every case the SNPs were not uniformly distributed on the DH10B chromosome (Figure S2). The sequence data from one of the multidrug-resistant (MDR) pools of isolates with extremely high fluoroquinolone MICs (H01) diverged greatly (>25%) from all the reference genomes, suggesting that, although they were classified metabolically as *E. coli*, genotypically they pushed the species boundary and represented an intriguing direction of future research. We excluded this pool from further SNP analysis.

Approximately 80% of SNPs detected in fluoroquinolone-resistant pools were in coding regions, located in 92% of the 4,357 annotated genes of DH10B (Figure S3a). Unanimous SNPs accounted for 64,773 loci. Despite the differences in numbers of isolates in the pools, the number of unanimous SNP calls in each pool did not correlate with the pool size (Figure S3b). This finding indicates that the clustering algorithm effectively put similar isolates together and demonstrates the usefulness of sequencing relatively large pools of bacterial genomes.

The environmental isolate, SMS-3-5 [GenBank: CP000970.1], is resistant to many antibiotics and has the highest MICs reported for several of these drugs [27]. The SMS-3-5 genome is diverged and larger than the K12 genome (Figure S4). We hypothesized that the genomes of multidrug-resistant clinical isolates would resemble SMS-3-5. Compared to the 5.1 Mb SMS-3-5 genome, we identified SNPs on 1,450,796 loci among the 15 remaining pools. In general, pools containing the fluoroquinolone-susceptible, non-MDR isolates exhibited more SNPs (107,395 loci in S01; 94,543 in S02) than MDR pools containing isolates with high fluoroquinolone MICs (81,403 in H03; 88,677 in M11), consistent with the hypothesis that the genomes of multidrug-resistant isolates resemble SMS-3-5.

**Figure 1:**
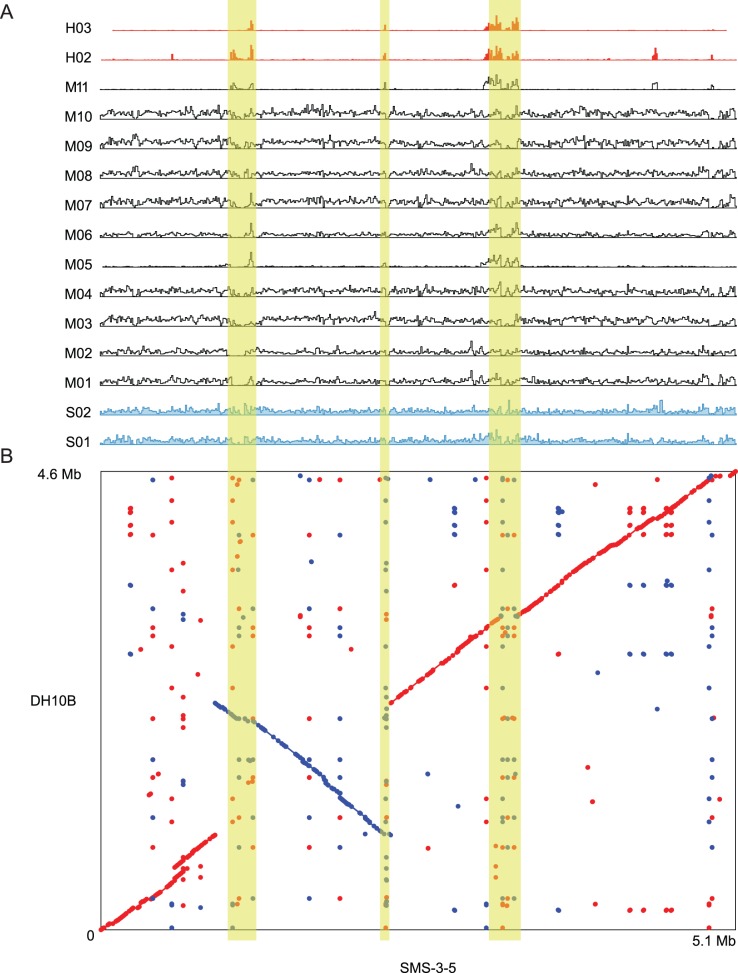
Pools of multidrug-resistant *E. coli* clinical isolates are genotypically similar to SMS-3-5. **A)** The distribution of SNPs in each pool along the chromosome was plotted against the SMS-3-5 reference genome. The *y*-axis for each pool represents a frequency of SNPs within binned regions of the chromosome. The height of the peaks was maximized to allow visualization of SNPs and does not imply a value of frequency relative to other pools. Peaks in the plot represent regions of high relative variability for a pool. “S” pools are shown in blue; “H” pools in red; “M” pools in black. **B)** Dot plot of DH10B and SMS-3-5. The DH10B and SMS-3-5 genomes were aligned using MUMmer. The SNP distribution plots for each pool were aligned to the dot plot in (A). Red dots show aligned contiguous sequences; blue dots show inversions. Highlighted in yellow are regions of consistent high SNP distribution across all pools.

Using the SMS-3-5 genome as the scaffold, SNPs detected in most of the pools were fairly uniformly distributed, but the pools M05, M11, H02, and H03 were clustered at specific loci (Figure 1a). A large-scale inversion [27] and duplications account for the major differences between the DH10B and SMS-3-5 genomes (Figure 1b). The regions of high SNP variation clustered near the breakpoints of these major chromosomal alterations (highlighted regions in Figure 1a), and thus may reflect regions of genomic instability. The same large chromosomal inversion can be found, in part, in at least one other strain from *E. coli* phylogenetic group D, the same group as SMS-3-5 [35]. This inversion, however, is not present in any of the other phylogenetic groups (Figure S5).

**Figure 2:**
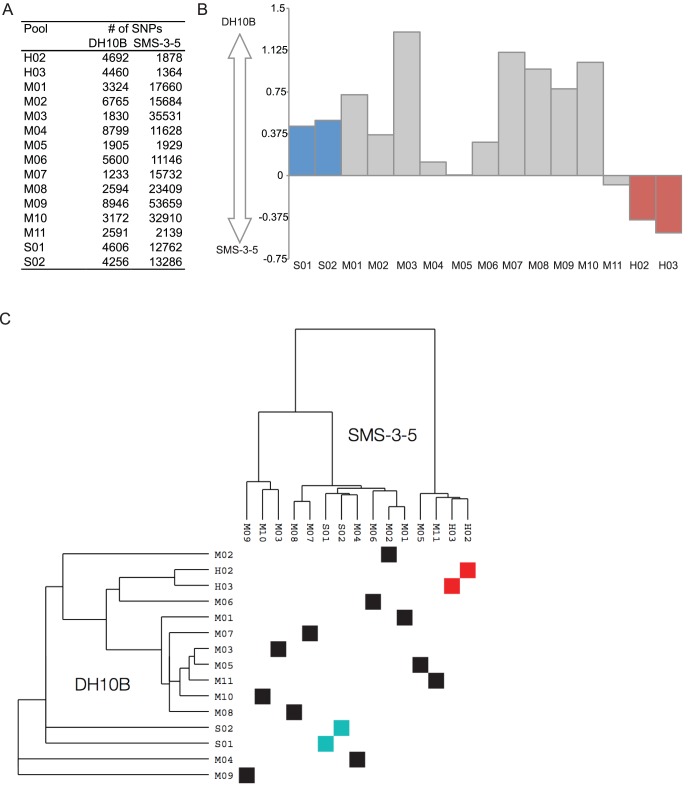
Relationship of pool consensus sequences to susceptible genome DH10B and resistant genome SMS-3-5. **A)** Total numbers of unanimous SNPs in each pool relative to DH10B and SMS-3-5. **B)** The similarity of each pool to DH10B and SMS-3-5. The logarithm of (unanimous SNPs relative to SMS-3-5/unanimous SNPs relative to DH10B) was plotted for each pool. Blue bars are “S” pools; red bars are “H” pools; gray bars are “M” pools. **C)** Dot plot of genomic similarity for each pool. The genomic similarity of each pool was calculated relative to each DH10B and SMS-3-5, resulting in a hierarchical clustering of the pools. Blue squares are “S” pools; red squares are “H” pools; black squares are “M” pools.

The number of SNPs called is a direct measure of the difference between that pool and the reference. Thus the log ratio of the number of unanimous pool-specific SNPs relative to either DH10B or SMS-3-5 (Figure 2a) reveals the similarity of each pool to the reference genomes (Figure 2b). The genomes of the multidrug-resistant clinical isolates with high fluoroquinolone MICs in the H02 and H03 pools were more similar to the environmental isolate than to DH10B. Likewise, the genomes in the drug-susceptible pools were more similar to DH10B.

We refined the SNP analysis by comparing the number of unanimous SNPs in common between any two pools relative to every other pool. The commonality of SNPs is a similarity metric that defines a distance map among pools. This similarity can be represented as a hierarchical tree (Figure 2c). The branch length of each tree in the axes denotes their unrooted relative distance in the hierarchical clustering process. The dot plot compares the hierarchical clustering of the pools using SNPs called against DH10B or SMS-3-5. The pools that were the most drug-susceptible clustered together and pools that were the most drug-resistant clustered together, regardless of which reference genome was used, again illustrating the relative independence of the pool-based sequencing to the reference genomes. One pair of multidrug-resistant pools (M5 and M11) also clustered together, indicating genotypic similarity. The dispersed nature of the remaining pools suggests that the key genotypic differences for antibiotic resistance and susceptibility involve different genomic regions in *E. coli*. This idea is consistent with the differences in distribution of SNPs, which were dependent on the scaffold used (Figure 1a). Phenotypic differences between the two genomes, therefore, are not a consequence of variations in a core set of genes, such as antibiotic target or resistance-encoding genes.

About half of all the unanimous SNPs were specific to a single pool (Figure S3c). The remaining unanimous SNPs were shared between at least two pools; 1.6% of which were found in all 13 pools of fluoroquinolone-resistant pools. Among all unanimous SNPs, homotypic changes (purine to purine or pyrimidine to pyrimidine) were detected twice as frequently as heterotypic SNP conversions, in agreement with most [36], [37], but not all [36] metazoan and human data. SNPs in metazoan genomes are usually biallelic (two variants per locus), attributed to the relative rarity of single nucleotide mutations and the long generation times of these organisms. Given the short generation times and active DNA metabolism of bacteria, we expected a higher rate or triallelism or even tetraallelism in polymorphic loci in our dataset. We discovered, however, that biallelic loci accounted for 99.2% of the SNPs we measured, with a scant few (0.8%) triallelic. At no single position were all four nucleotide variants measured.

### Novel SNPs Linked to Fluoroquinolone Resistance Represent a Potential Resistance Fingerprint

With the high frequency of biallelism we observed, SNPs from different phenotypic pools could be subtracted directly to reveal a potential genetic signature specific to a selected phenotype. We created a computational platform that performs arbitrary set arithmetic between the pools based on their phenotypes to enrich for alleles that are linked to a specific phenotypic character. From these sets of loci, we determined the annotated genes affected by non-synonymous changes, and established their commonality between the reference genomes to leverage the preexisting annotations. To our knowledge, this is the first time SNP subtraction has been carried out on pooled data. This approach can be generalized to enrich for SNPs linked with any phenotype for which large enough sequence data exist. As our dataset derives from a collection best studied for fluoroquinolone resistance [20], [21], [28], we performed the subtraction designed to enrich for alleles associated with this trait.

**Figure 3:**
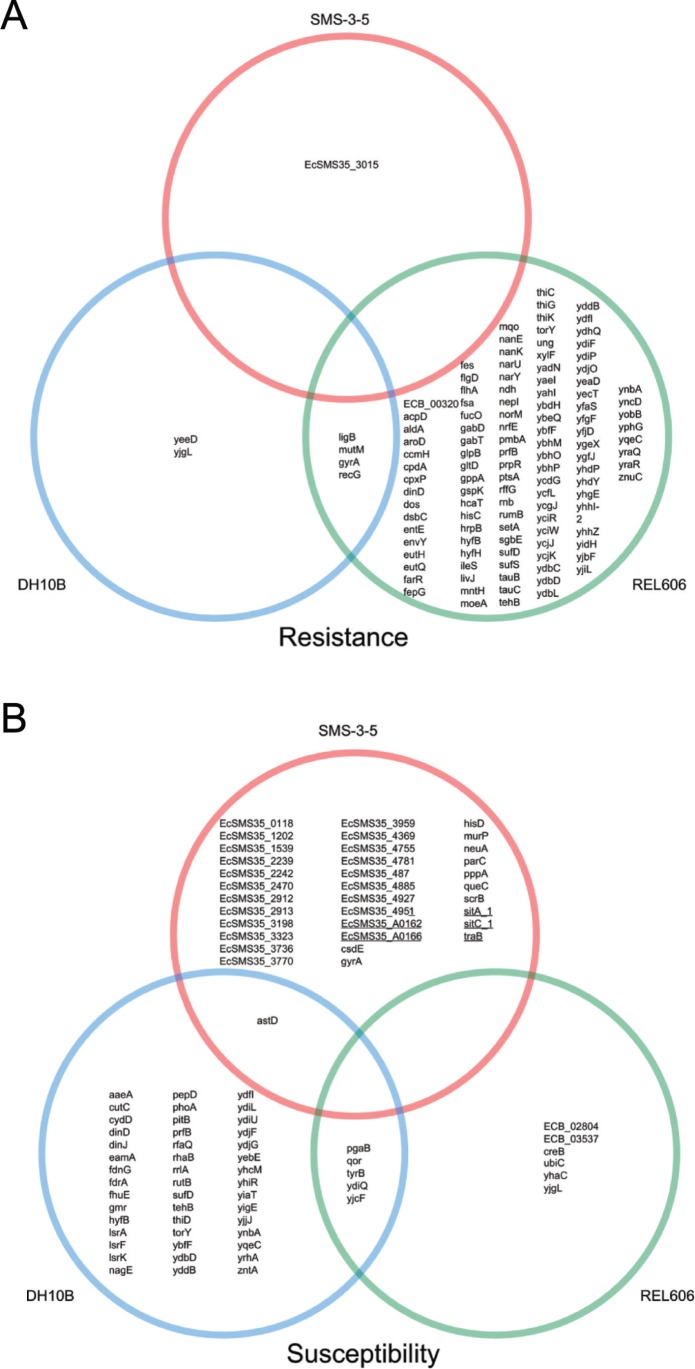
SNPs associated with fluoroquinolone resistance and susceptibility. Unanimous SNPs that result in non-synonymous changes in genes were computed relative to each of the three reference genomes, SMS-3-5 (multidrug-resistant with high fluoroquinolone MICs), DH10B, and REL606 (both antibiotic susceptible). Genes containing these variants for each reference genome are shown in Venn diagrams. **A)** Genes with allelic variants that were enriched in the fluoroquinolone-resistant phenotype. Variants that were called in any fluoroquinolone-susceptible pool were subtracted from those found in common among all fluoroquinolone-resistant pools. **B)** Genes with allelic variants that were enriched in the fluoroquinolone-susceptible phenotype. Underlined genes are encoded on an SMS-3-5-specific plasmid (pSMS35_130).

SNPs that occurred in either of the fluoroquinolone-susceptible pools (S01 and S02) were subtracted from the SNPs conserved in all 13 fluoroquinolone-resistant pools to reveal SNPs enriched for fluoroquinolone resistance (Figure 3a). Relative to the genome of DH10B, 230 unanimous SNPs in coding and noncoding regions were shared among all the fluoroquinolone-resistant pools, but were not unanimous in the fluoroquinolone-susceptible pools. Six of these SNPs were predicted to make non-synonymous changes to annotated genes (Figure 3a, genes contained within the blue circle). Against the REL606 genome, 989 SNPs conformed to the same fluoroquinolone-resistance-based criteria; 117 of these were non-synonymous (Figure 3a, genes listed within the green circle). Relative to SMS-3-5, one SNP associated with fluoroquinolone resistance was unanimous and corresponded to a non-synonymous change in the gene, EcSMS35_3015. When aligned using Gene Stream (http://xylian.igh.cnrs.fr/bin/align-guess.cgi), this gene shared 98.6% nucleotide sequence identity with the *ygfO* gene of DH10B, which encodes a xanthine/uracil permease. This SNP may be an important distinction between the fluoroquinolone-resistant clinical isolates and the environmental isolate.

To generate a SNP signature for fluoroquinolone resistance without biasing toward any single reference genome or any single *E. coli* strain, we only considered alleles that were in agreement with SMS-3-5, but deviated from both DH10B and REL606 (Figure 3a, genes contained within the blue (DH10B) and green (REL606) circles). Gene exclusivity from the Venn diagram could result from variations linked to the phenotype of the specific reference, or could be a consequence of a gene being absent from the annotation of either of the two other reference genomes. Conversely, inclusion of a gene in the Venn diagram strongly suggests some sort of involvement with fluoroquinolone resistance. We identified SNP variants of *gyrA*, *ligB*, *mutM*, and *recG* genes linked to fluoroquinolone resistance. The well-known L83 allele of *gyrA* exists in all of the fluoroquinolone-resistant isolates in this collection [20], and is the most common variant seen in fluoroquinolone-resistant *E*. *coli* and corresponding mutations in other gram-positive and gram-negative bacteria (reviewed in [38]). Identifying this *gyrA* allele supports the hypothesis that our subtraction method reveals SNPs associated with fluoroquinolone resistance.

The other three coding loci with SNPs discovered by this subtraction method correspond to non-synonymous variations in *recG* (A109T), *mutM* (A422S), and *ligB* (T127A), alleles that have never before been associated with fluoroquinolone resistance. Sanger sequencing confirmed the SNP variants in a subset of isolates representing all 15 pools (Table S3). For all three SNP variants, the variant was found in all resistant isolates and in several, but not all susceptible isolates. The difference in frequency with which these SNP variants were found in resistant pools compared to susceptible pools was significant by Chi-square test. Confirmation of these SNPs within individual isolates also validated that our gene annotation algorithms were unbiased toward any single reference.

### Novel SNPs Linked to Fluoroquinolone Resistance Demonstrate a Survival Phenotype under Genotoxic Stress

**Figure 4:**
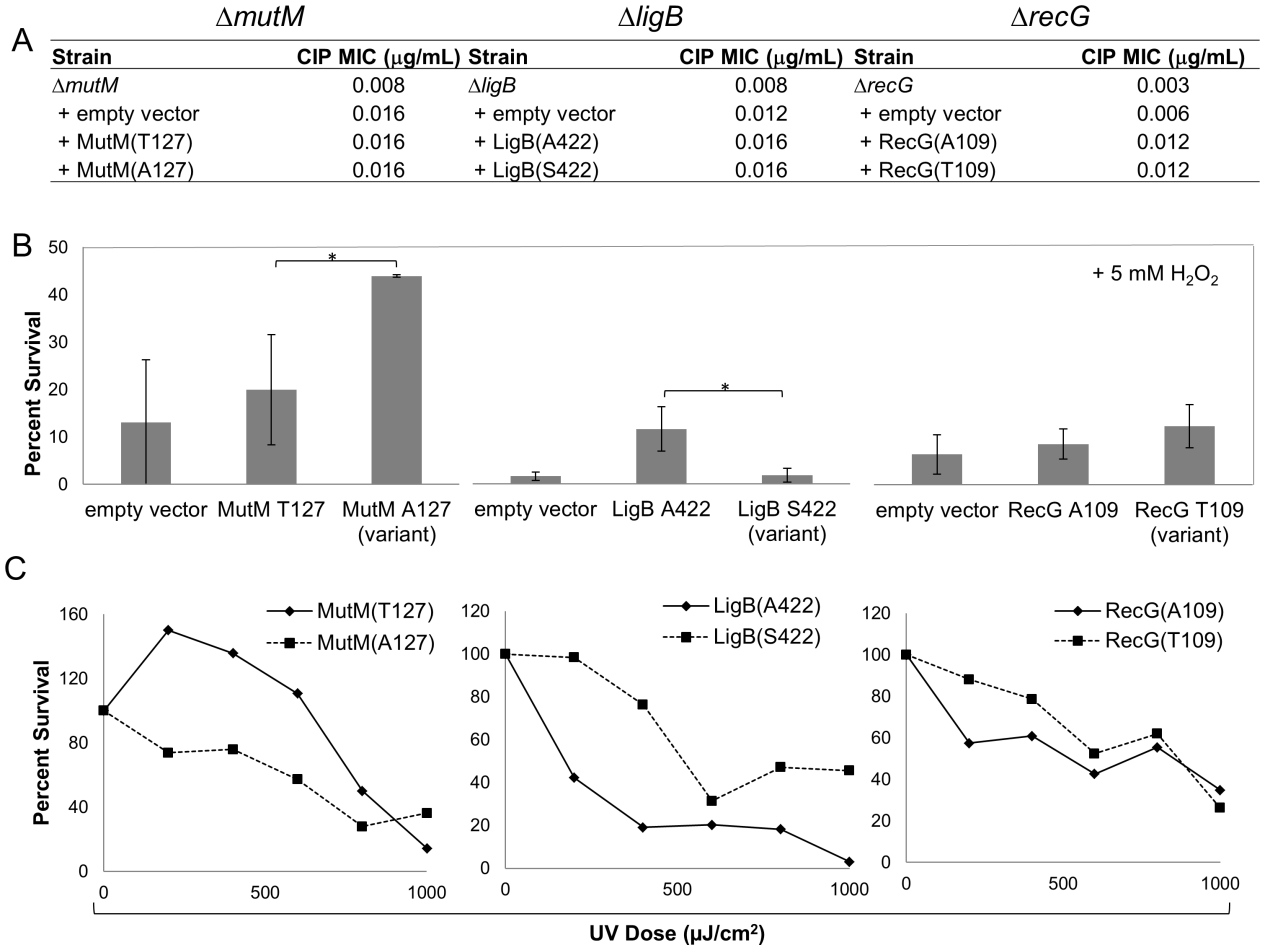
Effect of variants on ciprofloxacin, hydrogen peroxide, or ultraviolet light susceptibility. **A)** Ciprofloxacin (CIP) susceptibility. MICs were measured using E-test. Experiments were repeated twice with identical results. The CIP MIC for the parent strain was 0.016 µg/ml. **B)** H_2_O_2_ susceptibility. Cells were grown and exposed to 5 mM H_2_O_2_ for 20 minutes, then spread onto LB agar. Colonies were counted after overnight incubation at 37°C. Shown are the averages for three independent experiments. *p-value of <0.05. **C)** UV susceptibility. Cells were spread onto LB agar and exposed to various doses of UV light. Shown is a representative result of three separate experiments.

Deletion of *recG* increases fluoroquinolone susceptibility [39] and causes replication defects [40] in *E. coli.* To test whether these SNP variants contribute to fluoroquinolone resistance, we used the individual null mutants for *mutM*, *ligB*, and *recG* from the Keio knock-out strain collection [41]. Through sequencing, we found that the Keio parent strain matched DH10B in nucleotide identity at the *recG*, *mutM*, and *ligB* alleles. We then measured fluoroquinolone MICs for each mutant strain. Isogenic null mutants had ciprofloxacin MICs that were ≥2-fold lower than the parent (Figure 4a). For *mutM* and *recG*, plasmid-encoded complementation restored ciprofloxacin MICs to parent strain levels. The plasmid expressing *ligB* did not fully restore the MIC to that of the parent strain. Transformation of the parent strain with the *ligB* expression plasmid also lowered the ciprofloxacin MIC (Figure 4a).

The genes *ligB*, *mutM*, and *recG* all encode proteins known to interact with DNA, and both *mutM* and *recG* are known to be involved with the repair of DNA damage. Fluoroquinolone exposure results in cellular release of reactive oxygen species (ROS) and 8-oxoguanine nucleotides, which could cause significant damage to the chromosome [42]. It has been shown that a double deletion mutant of both *mutM* and *mutY* survives fluoroquinolone exposure better than the isogenic parent strain. This phenotype was attributed to a reduction in double-strand DNA breaks [43]. Fewer breaks in the chromosome and increased mutation rate could be advantageous in the presence of stress such as from fluoroquinolones. Given the role of MutM in repair of such damage, we tested whether the variants of LigB, MutM, and RecG responded differently to ROS. Plasmids encoding either gene variant were transformed into the respective null strains, and then exposed to genotoxic stress. When exposed to 5 mM H_2_O_2_, the fluoroquinolone-resistant associated variant of MutM (A127) survived ∼2 fold better than the T127 variant (Figure 4b). When exposed to ultraviolet (UV) radiation, the T127 variant survived better at low doses and the fluoroquinolone resistance-associated variant survived better at high doses (Figure 4c). The resistance-associated variant of MutM may lower the activity or affinity for 8-oxoguanine lesions in the chromosome, resulting in fewer breaks in the chromosome. The cell thus would have fewer breaks to repair than would the wild-type variant.

LigB has only a weak ligase activity *in vitro* [44]. If the resistance-associated LigB variant contributes to increased ligation activity, the cell could benefit under stress. We found that the resistance-associated LigB variant, S422, was ∼10 fold more susceptible to H_2_O_2_, but this same allele better survived UV radiation. There was no significant difference observed between the two RecG variants for survival with either H_2_O_2_ or UV stress. The *recG* allele differences may only become apparent when combined with the other alleles.

**Figure 5:**
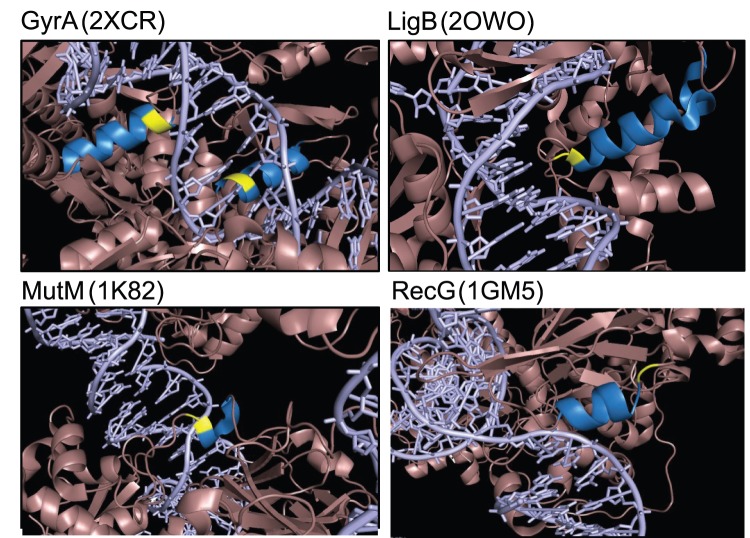
Position of variants in gyrase, LigB, MutM, and RecG co-crystal structures. The effect of amino acid substitutions was modeled using available co-crystal structures. GyrA PDB ID: 2XCR (from *Staphylococcus aureus*), LigA PDB ID: 2OWO *(*from *E. coli*), MutM PDB ID: 1K82 (from *E. coli*), and RecG PDB ID: 1GM5 (from *Thermotoga maritima*). Each amino acid variation (yellow), the α-helix affected (blue), and DNA (purple) are shown. For gyrase and MutM, the variants associated with drug-susceptible laboratory *E. coli* strains are shown. For LigB and RecG the variants tightly linked to fluoroquinolone resistance are shown.

To determine where within each protein the SNP variants were located, we searched for crystal structures in the Protein DataBase (PDB; http://www.rcsb.org/pdb/home/home.do). Co-crystal atomic structures with DNA exist for three of the four proteins (GyrA PDB ID: 2XCR; MutM PDB ID: 1K82; and RecG PDB ID: 1GM5). LigA was used to model LigB (LigA PDB ID: 2OWO). The amino acids impacted by the allelic variants are all located in alpha helices directly adjacent to a domain that interacts with DNA (Figure 5). Remarkably, the variants in *ligB*, *mutM*, and *recG* are all located at the “cap” of these alpha helices. Thus, the variable amino acid residues may affect protein interactions with DNA.

### SNPs Associated with Fluoroquinolone Resistance are Linked

The alleles of *gyrA*, *ligB*, *mutM*, and *recG* were conserved in all of our tested fluoroquinolone-resistant *E. coli* clinical isolates (Table S3). To determine how frequently these variants occur in other sequenced *E. coli*, we analyzed the 39 fully annotated, complete *E. coli* genomes archived in GenBank and the 83 annotated draft genomes from the Broad Institute *Escherichia coli* Antibiotic Resistance Sequencing Project, Broad Institute of Harvard and MIT (http://www.broadinstitute.org/). Ten genomes contained the *gyrA* L83 variant; nine of these genomes also contained all three of the novel resistance-linked variants of the genes. The tenth genome encoded the *ligB* and *recG* variants, but not the *mutM* variant. The *gyrA* L83 allele, then, is 100% linked to the *ligB* and *recG* alleles and 90% linked to all three. Such tightly linked inheritance implies a linked activity that is relevant to fluoroquinolone resistance. Of 122 strains in GenBank and Broad Institute, 109 encoded either none (15 strains) or all three (94 isolates) of the resistance-associated alleles. Of the remaining 13 isolates and strains, four encode the resistance-linked alleles of *ligB* and *recG*, four encode the *mutM* and *ligB* alleles, and five encode the *mutM* allele alone.

**Figure 6:**
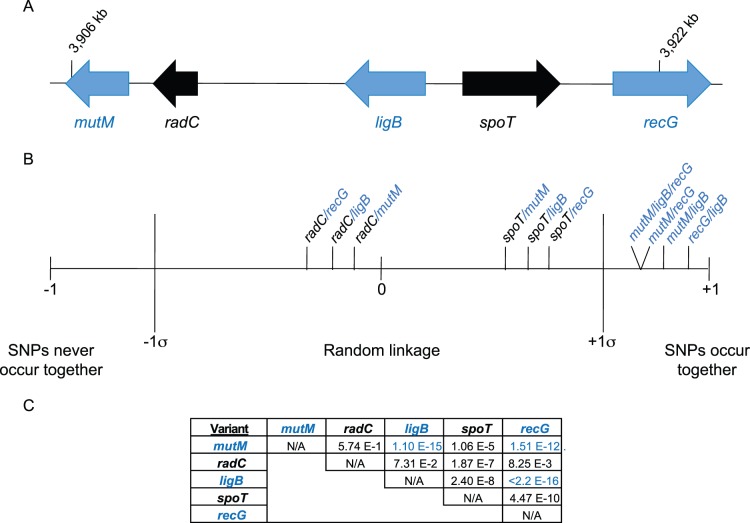
Linkage analysis of the *mutM*, *ligB*, and *recG* fluoroquinolone resistance associated SNPs. **A)** Relative chromosomal location of *mutM*, *radC*, *ligB*, *spoT*, and *recG* in *E. coli* DH10B. **B)** To determine the linkage frequency, 39 complete *E. coli* genomes in GenBank and 83 draft genomes from the Broad Institute were analyzed at the positions of variants in *mutM*, *ligB*, and *recG*. Allelic variants in *radC* and *spoT* were selected for relative comparison based on their location between the genes of interest. The linkage of each pair of allelic variants was normalized to a range from never linked (−1) to always linked (+1). The sigma (σ) denotes one standard deviation for a normal distribution. Strains with unannotated genes were not included in the analysis. **C)** The statistical significance of the linkage was calculated using two-by-two contingency tables and Χ^2^ analysis. With a Bonferroni correction for multiple comparisons, *p*-values <0.005 are considered significant. The statistical analysis was done using R version 2.12.2.

The *ligB*, *mutM*, and *recG* genes are encoded in a 15 kb cluster on the chromosome (Figure 6a). Linkage of alleles among these three genes could be explained by their close chromosomal proximity through co-inheritance or from selective pressure. If the three alleles are co-inherited through physical linkage, then all of the alleles within the cluster should be linked. We identified biallelic variants: one that occurs in *radC*, encoding a DNA repair protein, located between *mutM* and *ligB*; and *spoT,* encoding a (p)ppGpp synthase, located between *ligB* and *recG* (Figure 6a). We performed pair-wise comparisons across the genomes in GenBank and the Broad Institute. Comparison of either *spoT* or *radC* with *mutM*, *recG*, or *ligB* showed that these variants were not linked, and their probability of being found together was roughly equal to that of random chance (Figure 6b and Figure 6c). However, *mutM*, *recG*, and *ligB* were very tightly linked. This result suggests that physical proximity of the *mutM, ligB,* and *recG* loci is insufficient to explain the tight linkage of the alleles. Instead, the three alleles may be under a positive evolutionary selection. For example, they might cooperatively provide an adaptability advantage during exposure to antibiotics. Alternatively, it may be that the genes in this region were once physically linked, but the intervening genes were under different selective pressure to separate away from the other genes. Selective pressure of *mutM*, *ligB* and *recG* linkage seems more likely given that the pair-wise linkage of the interspaced genes approximated random chance.

### Novel SNP Variants Correspond to Fluoroquinolone Susceptibility

Above, following the traditional mindset of genetic alteration associated with disease, we analyzed SNPs associated with fluoroquinolone resistance. We wondered whether there might be SNPs associated with fluoroquinolone susceptibility. To identify potential genomic signatures linked to antibiotic susceptibility, we subtracted SNPs found in any of the “M” or “H” pools from unanimous SNPs in common between the two fluoroquinolone-susceptible pools (S01 and S02). The subtraction enriched for SNPs in 35 genes in common among fluoroquinolone-susceptible pools, DH10B, and REL606, but differed from SMS-3-5 (Figure 3b, genes listed in the red circle). Five of these genes are encoded on plasmid pSMS35_130 [GenBank: NC_010488.1], and the rest are on the chromosome. The identified EcSMS35-labeled genes encode sequences found in other published sequences but none correspond to any well-annotated genes.

In agreement with our previously published data [20], the S83 allele of *gyrA* and the S80 allele of *parC* were found only in fluoroquinolone-susceptible pools. These susceptible *gyrA* and *parC* alleles were ubiquitous among the fluoroquinolone-susceptible *E. coli* genomes in GenBank and the Broad Institute. Potential alleles associated with antibiotic susceptibility may be as useful as their resistant counterparts in the identification of a resistance phenotype and will be an interesting area for future exploration.

### Additional Variants in Topoisomerase Genes Cluster in Pools

In addition to the *gyrA*L83 variant, alleles of the *gyrA* gene (encoding amino acid Y/N87) and the *parC* gene (encoding I80 or K/G84) occur frequently in fluoroquinolone-resistant *E. coli* [10], [20]. Thus, we examined these alleles in the fluoroquinolone-resistant pools. Several pools contained the *parC*I80 variant as a mixed SNP. Although the *gyrA*Y/N87- and *parC*K/G84-encoding alleles were found in the pools containing fluoroquinolone-resistant isolates at high frequency, none were unanimous in any of the pools. Pool M03 contained 13 isolates that are resistant only to fluoroquinolones (Table 1). The *parC*I80 allele was unanimous in this pool. Assuming that the mutation rate of the *parC* gene does not differ from other loci and that the likelihood of the *parC*S80 and *parC*I80 variants is equal, finding this unanimous SNP in a pool of 13 isolates (1/2^13^ = 1 in 8,192) cannot be random chance. This result may suggest that resistance to fluoroquinolones alone requires this *parC* variant. The linkage of the *parC*I80 with a pool resistant solely to fluoroquinolones implies that, in the absence of resistance mechanisms to other antibiotics, the addition of the *parC*I80 variant to the *gyrA*L83 variant may be necessary for clinically-relevant fluoroquinolone MICs. Experiments using isogenic strains showed that *parC*G84 only affected fluoroquinolone MICs when combined with both *gyrA*L83 and *parC*I80; *parC*K84 alone, in fact, decreased fluoroquinolone MICs [8]. In our clinical isolate collection [20] and as far as we know for any *E. coli* clinical isolate [12], [45], *parC* mutations were never found alone.

### Cryptic Prophage Sequences in Clinical Isolates of *E. coli*

Wang *et al.* found that deletion of any of three cryptic prophages (rac, e14, and CP4-6) lowered nalidixic acid MICs in the *E. coli* BW25113 K-12 strain [46]. Despite very good mappability and the very high coverage of the pooled sequences, these prophage sequences were detected infrequently (Figure S6a). Direct PCR screening for *intR* for rac and *perR* for CP-4-6 verified that these sequences were found equally in fluoroquinolone-susceptible and fluoroquinolone-resistant clinical isolates (Figure S6b). Thus, in spite of affecting quinolone MICs, these prophage sequences are not correlated with fluoroquinolone resistance in clinical isolates.

## Discussion

Here, we report a novel GWAS analysis method using phenotype-based pooling, next generation sequencing, and SNP subtraction. The pooling approach, in addition to validating known variants (*gyrA* L83 and *parC* I80), can feasibly uncover genetic variants involved in a specific phenotype without the expense and challenge of sequencing and curating independent genome assemblies. Common mapping strategies assume that resequencing is done on descendant clones from the reference. We demonstrated that we can use indirectly related reference genomes as an annotation platform. While we used this method to identify novel alleles associated with fluoroquinolone resistance, we anticipate it could be used to probe for genomic commonalities associated with any phenotype.

The environment was implicated as a source of antibiotic resistance genes because of its diverse microbiota and the presence of antibiotics, both naturally produced and residual from agricultural use [23], [26]. The antibiotic resistome of soil bacteria bears high nucleotide identity to the same resistance genes within known clinical pathogens, providing strong evidence of the relatedness between these two bacterial populations [47]. We uncovered extraordinary genomic similarity between SMS-3-5 and the multidrug-resistant *E. coli* clinical isolate pools that had the highest fluoroquinolone MICs (M05, M11, H02, and H03). The similarity was not confined to a specific region or regions, *e.g*., a pathogenicity island or a plasmid, but was evenly spread across the entire genome, with the highest nucleotide variation occurring at the regions adjacent to inversion and duplication events in the ancestry of the reference strains.

Fluoroquinolones kill bacteria through the accumulation of topoisomerase-bridged DNA breaks and through the release of oxidative radicals that damage the chromosome [42], [48]. MutM removes mutagenic 8-oxoguanines in the genome after they have been incorporated as part of the bacterial GO system [49]. Deletion of MutM alone resulted in a modest increase in strain evolvability [50]. Deletion of the *mutM* and *mutY* genes together increased *E. coli* survival in the presence of antibiotics [43], and demonstrated a hypermutator phenotype in *Pseudomonas aeruginosa* [51]. As a consequence of the inability to repair DNA mismatches, bacteria accumulate genomic mutations. Clinically-isolated bacteria are frequently hypermutators. Though many of these mutations come at deleterious fitness costs, some mutations convey a selective advantage for occupying a niche within a host. Together with MutM, MutY prevents G:C to T:A transversions to protect the integrity of the chromosome [49]. The MutM variant we found, either alone or in combination with the LigB and RecG variants, may exhibit a hypermutator phenotype. If so, then perhaps the coordinated changes in these DNA modifying enzymes increase bacteria survival in response to the genotoxic stress created by fluoroquinolones. The mutation rate and evolvability of our identified SNP variants are topics of future research.

The modified residues of r*ecG, mutM,* and *ligB* each capped an alpha helix that is close in proximity to regions involved with DNA binding. The variants of RecG and LigB associated with fluoroquinolone resistance are predicted to stabilize the helices important for DNA binding. Stabilized forms of RecG and LigB may increase their ability to repair DNA damage induced by fluoroquinolones. Conversely, the MutM variant associated with fluoroquinolone resistance is predicted to destabilize the DNA binding helix, possibly by modifying nucleotide excision activity [43]. Taken together, these changes in *mutM*, *ligB* and *recG,* could set the stage for the *gyrA* L83 encoding mutation to occur.

We uncovered alleles in genes that potentially represent the fluoroquinolone susceptibility phenotype. The concept of genetic variations associated with antibiotic susceptibility is distinct from the conventional view, where susceptibility is a default state upon which drug resistance variations are layered. By considering such a possibility, we found both known (such as those in *gyrA* and *parC*) and novel alleles that can be linked to fluoroquinolone susceptibility. Maintaining drug susceptibility alleles in microflora could be an important way to combat the rise of drug resistance. The measure of frequency of alleles associated with antibiotic resistance or antibiotic susceptibility comprises a genomic signature that could be exploited in a diagnostic system to rapidly determine the antibiotic resistance of a pathogen. Such a system would minimize empirical antibiotic prescription, maximize treatment efficacy, and extend the useful life of the current antibiotic arsenal.

## Materials and Methods

### Materials and Reagents

Mueller-Hinton (MH) broth was from Difco (Sparks, MD); tryptone and yeast extract were from Becton Dickinson and Company (San Jose, CA); PureLink™ Pro 96 Genomic DNA Kit was from Invitrogen (Carlsbad, CA). NanoDrop® Spectrophotometer ND-1000 was from Thermo Scientific (Wilmington, DE).

### Sequencing Pool Design

Hosital-derived antibiotic susceptibility data (“S” for sensitive, “I” for intermediate, and “R” for resistant) for 17 antibiotics (listed in Table S1) and our quantitative susceptibility data for four fluoroquinolones (MICs for ciprofloxacin (CIP), gatifloxacin (GAT), levofloxacin (LVX), and norfloxacin(NOR)), were used to group 214 representative clinical isolates into pools by *k*-means clustering using Cluster 3.0 [29]. Strings of such data (*e.g.,* R-S-I-R-R-S-400-10, *etc*) were used to represent each isolate in the *k*-means clustering. 32 pools were generated by the clustering algorithm. Following manual inspection, pools were removed if they contained only one strain or if they were highly similar to other pools, but lacked sufficient qualitative susceptibility data. Sixteen pools containing a total of 164 strains (Table 1) were submitted for whole-genome sequencing. The phenotypes represented in these pools ranged from susceptible to all drugs tested to nearly pan-drug resistant. We designated the pools as “M” when the isolates therein had fluoroquinolone MICs greater than the resistance breakpoints (ciprofloxacin ≥4 µg/ml, gatifloxacin and levofloxacin ≥8 µg/ml and norfloxacin ≥16 µg/ml) but less than 400 µg/ml, and as “H” when fluoroquinolone MICs exceeded 400 µg/ml.

### Genomic DNA Isolation and Pool Assembly

Genomic DNA was isolated from each isolate using the PureLink™ Pro 96 Genomic DNA Kit and quantified using a NanoDrop® Spectrophotometer ND-1000. All DNA samples had A_260_/A_280_ greater than 1.8. Genomic DNA was pooled according to the results of the clustering analysis such that each genome was represented at equimolar concentrations within a pool.

### SOLiD™ Sequencing

Pools of genomic DNA were sequenced using 2×25 bp mate-paired libraries with the Applied Biosystems SOLiD™ System (Life Technologies). Briefly, 16–45 µg of DNA per library were sheared to 2.0 kb using the Covaris™ S2 System according to the manufacturer’s instructions. Genomic EcoP15I restriction enzyme sites were methylated prior to EcoP15I CAP Adaptor ligation. Samples were then size-selected and circularized incorporating the internal adaptor. In the subsequent EcoP15I restriction enzyme step, the DNA was cleaved 25–27 bp away from the unmethylated enzyme recognition site in the CAP adaptor forming the DNA mate-pair. Finally, P1 and P2 adaptors were ligated to the mate-paired libraries for PCR amplification. Each library template was clonally amplified on SOLiD P1 beads using emulsion PCR. Templated (P2 positive) beads were then enriched and deposited on an octet of a slide. SOLiD sequencing was carried out at 2×25 bp, using SOLiD v3.5 chemistry according to the manufacturer’s instructions.

### Sequence Analysis

Sequenced reads were mapped to selected genomes using BioScope v 1.2.1. 2×25 mate-pair reads were mapped to the reference genome allowing for up to two mismatches per read. The alignment with the highest mapping quality value was chosen as the primary alignment. If two or more alignments had the same score then one of them was randomly chosen as the primary alignment. Reads were then paired, allowing up to four mismatches across the pair by searching for pairs which are in the expected orientation and insert size (BioScope v 1.2.1). SNP calling was performed using the BioScope diBayes algorithm, which incorporates position and probe errors as well as color quality value information for SNP calling, at medium-stringency setting (http://www3.appliedbiosystems.com/cms/groups/mcb_marketing/documents/generaldocuments/cms_057817.pdf). DiBayes filtered reads with mapping quality <8. DiBayes with medium stringency setting needs 2x to call homozygous and 3x to call a heterozygous SNP. Annovar [52] was used to annotate the SNPs using gene lists from NCBI for the three platform genomes. Custom scripts were written in Perl for subsequent filtering of SNPs, and positions were further processed using Cluster [53], Java TreeView (http://jtreeview.sourceforge.net/), Sequencher (http://www.genecodes.com), and DataGraph (http://www.visualdatatools.com/DataGraph/). GO analysis was performed using the DAVID algorithm [54] and reference genome alignments were done on MUMmer [55].

### Sequence Comparison

A secure HBase database housed all the SNP information and a custom Java program mined the collection of SNPs. This program sought SNPs that occurred only in either fluoroquinolone-susceptible or fluoroquinolone-resistant clinical isolates compared within each reference genome. A total of 709,163 unique SNPs from all the pools to reference comparisons were analyzed. Where potential fluoroquinolone-specific correlations did occur, the position and related information, including change, gene, pool identifier, and type of change were saved for downstream analysis.

### Atomic Structure Prediction

Hydrogen atoms were placed on all polar heavy atoms in the crystal structures and their positions optimized using the modeling software Rosetta [56]. Stabilization interactions involving helix-capping motifs were identified if the corresponding residues established side chain/backbone hydrogen bonds of energies lower than 0.5 kcal/mol.

### Overexpression Plasmid Construction

Strains from the ASKA library [57] harboring plasmids containing the genes *recG, ligB,* and *mutM* from MG1655 *E. coli* were obtained from the National Institute of Genetics, Shizuoka, Japan. Site directed mutagenesis of single amino acids was performed using the QuikChange protocol (Agilent) using the following primers. *recG*: 5′-CAATGAAAAATAGCCTGGCGACGGGCCGCCGTGTAC-3′ and 5′-GTACACGGCGGCCCGTCGCCAGGCTATTTTTCATTG-3′. *ligB*: 5′-CGCTTAGTCTGGCTGGGGTCAAAACAGGTTCTTGGG-3′ and 5′- CCCAAGAACCTGTTTTGACCCCAGCCAGACTAAGCG-3′. *mutM*: 5′-GGAAGGGCATAATGTGCTGGCCCATCTTGGACCGG-3′ and 5′-CCGGTCCAAGATGGGCCAGCACATTATGCCCTTCC-3′. After point mutations were made and verified using Sanger sequencing, the expression plasmids containing each genomic variant were transformed separately into the corresponding Keio knockout mutant by standard methods.

### Measurement of MICs

Ciprofloxacin MICs were determined using E-Test® strips (Biomérieux, Durham, NC). Measurements were made according to the manufacturer’s instructions. Isogenic mutants were from the Keio knockout collection [41].

### H_2_O_2_ and UV Radiation Survival Assays

Overnight cultures of the various deletion strains and those strains each complemented with the two different alleles were diluted 100-fold into 10 ml of fresh Luria-Bertani (LB) medium containing 30 µg/ml (final concentration) chloramphenicol (Sigma, St. Louis, MO), when necessary. These cultures were incubated, with shaking, at 37°C to mid-logarithmic phase (OD_600_ = 0.4). Cultures were then divided into two 5 ml aliquots and H_2_O_2_ was added to a final concentration of 5 mM to one aliquot. Samples were incubated for 20 minutes and treated with an excess (50 µg/ml) of catalase (Worthington Biochemical, Lakewood, NJ). Cultures were serially diluted, plated on LB agar, and incubated overnight at 37°C. Colony forming units (CFUs) were enumerated after 16 hours. Each experiment was repeated at least three times.

For the UV irradiation experiments, cultures were grown as previously and serially diluted after reaching an OD_600_ = 0.4. A 100 µl aliquot of the 10^−5^ dilution (∼3×10^3^ cells) was spread on LB agar. Plates were dried at room temperature for 20 minutes, exposed to the indicated doses of UV light using a CL-1000 ultraviolet crosslinker, and incubated overnight at 37°C. CFUs were counted after 16 hours. Each experiment was repeated at least three times.

## Supporting Information

Figure S1 Experimental design workflow. Selected isolates were clustered into pools based on their antibiotic resistance. Genomic DNA was pooled and sequenced on the SOLiD 3 platform. Sequence reads were mapped to using genomes DH10B (susceptible), REL606 (susceptible) and SMS-3-5 (multidrug-resistant) as platforms. Unanimous SNP variants were called for each pool. For nonsynonymous alterations, the genes were identified. Genetic variation associated with fluoroquinolone resistance were those unanimous in all resistant pools, but not called in any fluoroquinolone-susceptible pools. Variations associated with fluoroquinolone susceptibility were those unanimous in the fluoroquinolone-susceptible pools, but not called in any fluoroquinolone-resistant pool.
(TIF)

Figure S2 SNP frequency plots relative to the DH10B genome. The frequency of unanimous SNPs in each pool was plotted along the DH10B chromosome. The *y*-axis for each pool was altered to allow visualization of SNPs and does not imply a value of frequency relative to other pools. Peaks in the plot represent regions of high variability for a pool. “H” pools are shown in red; “S” pools are in blue.
(EPS)

Figure S3 Genome-wide analysis of unanimous SNPs called relative to DH10B. **A)** The percentage of genes containing variations associated with fluoroquinolone resistance. For each of the 13 fluoroquinolone-resistant pools, SNPs in genes were mapped to the DH10B genome. SNPs that were the same in identity and position were denoted as unanimous SNPs. SNPs that failed to match both of these criteria were named mixed SNPs. **B)** Occurrence of unanimous SNPs as a function of pool size. The total number of unanimous SNPs detected in each pool relative to DH10B was plotted versus pool size. Pool H01 was dropped from further analysis as an outlier by the Extreme Studentized Deviate test statistic. A best-fit line to the data for the remaining 13 resistant pools showed a weak negative correlation (R^2^ = 0.0907), which was not statistically significant. **C)** Distribution of fluoroquinolone resistance-specific SNPs. SNPs found in any of the fluoroquinolone-susceptible pools were subtracted from the set. The distribution of the resistance-specific, unanimous SNPs is shown.
(EPS)

Figure S4 Phylogenetic analysis of published *E. coli* genomes. A phylogenetic analysis of the 33 *E. coli* genomes in GenBank showed SMS-3-5 (top row) is the most diverged. The heat map color range shows the relative number of additional sequence reads from each pool that could be mapped to each reference genome. (Blue = fewest; Red = most). A maximum number of additional sequence reads could be mapped to SMS-3-5.
(EPS)

Figure S5 Dotplots comparing SMS-3-5 to other *E. coli* genomes. **A)** Dotplot comparing the DH10B and W3110 genomes. The SMS-3-5 inversion is not present. **B)** Dotplot comparing SMS-3-5 and W3110. **C–H)** Dotplots comparing the SMS-3-5 genome to the genomes of two strains from each of the phylogenetic groups B2 **(C, D)**, D **(E, F)** and B1 **(G, H)**. The inversion is found only in SMS-3-5 with the exception of IAI39 **(E)**, which has a smaller inversion in the same region. All dotplots were made using Geneious software. Accession numbers are as follows: W3110 [GenBank: AP009048.1]; UTI89 [GenBank: CP000243]; NA114 [GenBank: CP002797]; IAI39 [GenBank: CU928164]; UMN026 [GenBank: CU928163]; SE11 [GenBank: AP009240]; and IAI1 [GenBank: CU928160].
(EPS)

Figure S6 Representation of prophage in *E. coli* clinical isolates. **A)** Prophage coverage in pools. The short reads of each pool were probed for the presence of cryptic prophage. Coverage (*y*-axis) was normalized to the average coverage of DH10B core genes and displayed by intensity for each prophage in each pool (black, low coverage; red, high relative coverage). **B)** Presence of prophages in fluoroquinolone-susceptible and fluoroquinolone-resistant clinical isolates. Individual isolates were probed for the prophage genes *intR* for rac and *perR* for CP4-6 by PCR. The percentage of isolates testing positive for each gene is shown for all fluoroquinolones-susceptible (n = 18) and fluoroquinolone-resistant (n = 65) isolates tested. These isolates were selected to represent all pools shown in (A).
(EPS)

Table S1 Antibiotics classes.
(DOCX)

Table S2 Patient demographics and clinical isolate culture sites.
(DOCX)

Table S3 Confirmation of allelic variants by Sanger sequencing.
(DOCX)
