# Correction: Novel Conserved Genotypes Correspond to Antibiotic Resistance Phenotypes of *E. coli* Clinical Isolates

**DOI:** 10.1371/annotation/d1a6c01c-b690-41a1-86e2-2142ff02cb1e

**Published:** 2014-01-22

**Authors:** Michelle C. Swick, Michael A. Evangelista, Truston J. Bodine, Jeremy R. Easton-Marks, Patrick Barth, Minita J. Shah, Christina A. Bormann Chung, Sarah Stanley, Stephen F. McLaughlin, Clarence C. Lee, Vrunda Sheth, Quynh Doan, Richard J. Hamill, David Steffen, Lauren B. Becnel, Richard Sucgang, Lynn Zechiedrich

A funding organization for the 13th author, Richard Hamill, was incorrectly omitted from the Funding Statement. The following sentence should be included with the Funding Statement: 

"RJH was supported in part by resources provided by the Department of Veterans Affairs."

In addition, an affiliation for the 13th author was incorrectly omitted from the byline. Richard Hamill is also affiliated with The Michael E. DeBakey VA Medical Center, Houston, Texas, United States of America.

