# Supplementary figures and images for: Novel Conserved Genotypes Correspond to Antibiotic Resistance Phenotypes of E. coli Clinical Isolates
Source: PLoS One. 2013 Jun 18;8(6):e65961. doi: 10.1371/journal.pone.0065961 (PMC3688849; doi:10.1371/journal.pone.0065961)

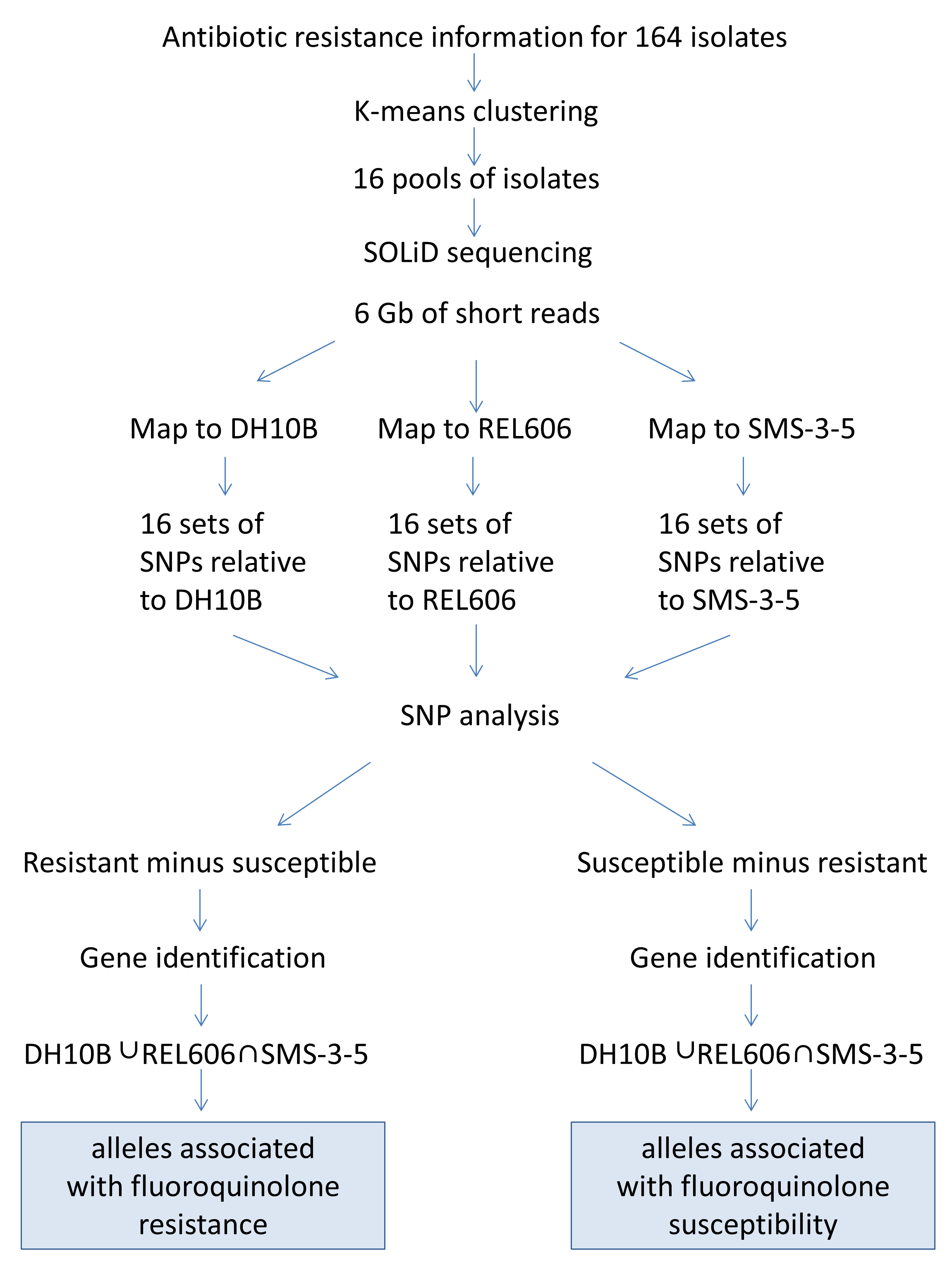

Supplement: Figure S1 — Experimental design workflow. Selected isolates were clustered into pools based on their antibiotic resistance. Genomic DNA was pooled and sequenced on the SOLiD 3 platform. Sequence reads were mapped to using genomes DH10B (susceptible), REL606 (susceptible) and SMS-3-5 (multidrug-resistant) as platforms. Unanimous SNP variants were called for each pool. For nonsynonymous alterations, the genes were identified. Genetic variation associated with fluoroquinolone resistance were those unanimous in all resistant pools, but not called in any fluoroquinolone-susceptible pools. Variations associated with fluoroquinolone susceptibility were those unanimous in the fluoroquinolone-susceptible pools, but not called in any fluoroquinolone-resistant pool. (TIF) [file pone.0065961.s001.tif]
