# Supplementary material for: Novel Conserved Genotypes Correspond to Antibiotic Resistance Phenotypes of E. coli Clinical Isolates
Source: PLoS One. 2013 Jun 18;8(6):e65961. doi: 10.1371/journal.pone.0065961 (PMC3688849; doi:10.1371/journal.pone.0065961)
Supplement: Table S1 — Antibiotics classes. (DOCX) [file pone.0065961.s007.docx]

**Table S1. Antibiotics classes.**

| Antimicrobial Class | Antibiotic |
| --- | --- |
| **Fluoroquinolones** | Norfloxacin |
|  | Ciprofloxacin |
|  | Gatifloxacin |
|  | Levofloxacin |
| **Aminoglycosides** | Amikacin |
|  | Gentamicin |
| **Cephalosporins** | Ceftriaxone |
|  | Ceftazidime |
|  | Cefotetan |
|  | Cefepime |
|  | Cefoxitin |
|  | Cefotaxime |
|  | Cefazolin |
| **Monobactams** | Aztreonam |
| **Carbapenems** | Imipenem |
| **Combination Penicillins** | Amoxicillin-clavulanic acid |
|  | Piperacillin-tazobactam |
|  | Ticarcillin-clavulanic acid |
| **Nitrofurans** | Nitrofurantoin |
| **Penicillins** | Ampicillin |
| **Combination Synthetics** | Trimethoprim-sulfamethoxazole |
