# Supplementary material for: Novel Conserved Genotypes Correspond to Antibiotic Resistance Phenotypes of E. coli Clinical Isolates
Source: PLoS One. 2013 Jun 18;8(6):e65961. doi: 10.1371/journal.pone.0065961 (PMC3688849; doi:10.1371/journal.pone.0065961)
Supplement: Table S2 — Patient demographics and clinical isolate culture sites. (DOCX) [file pone.0065961.s008.docx]

**Table S2. Patient demographics and clinical isolate culture sites.**

| Culture Site | n* | | (%) | | |
| --- | --- | --- | --- | --- | --- |
| Urine | 133 | | (84.7) | | |
| Blood | 9 | | (5.7) | | |
| Sputum | 4 | | (2.5) | | |
| Nose | 1 | | (0.6) | | |
| Kidney abscess | 2 | | (1.3) | | |
| Abdomen | 2 | | (1.3) | | |
| Sacral tissue | 2 | | (1.3) | | |
| Wound | 1 | | (0.6) | | |
| Exudate | 1 | | (0.6) | | |
| Bronchial lavage | 1 | | (0.6) | | |
| Biliary tract | 1 | | (0.6) | | |
| **Gender** | | **n*** | | **(%)** |  |
| Male | | 58 | | (36.9) |  |
| Female | | 99 | | (63.1) |  |
| **Age** | | 6-90 | | (mean = 53) |  |

*7 isolates did not have patient data
