# Supplementary material for: Novel Conserved Genotypes Correspond to Antibiotic Resistance Phenotypes of E. coli Clinical Isolates
Source: PLoS One. 2013 Jun 18;8(6):e65961. doi: 10.1371/journal.pone.0065961 (PMC3688849; doi:10.1371/journal.pone.0065961)
Supplement: Table S3 — Confirmation of allelic variants by Sanger sequencing. (DOCX) [file pone.0065961.s009.docx]

**Table S3. Confirmation of allelic variants by Sanger sequencing.**

| Pool | No. of isolates tested/ Total isolates in pool | Isolates containing the allele associated with resistance | | |
| --- | --- | --- | --- | --- |
|  |  | ***ligB* (%)** | ***recG* (%)** | ***mutM* (%)** |
| S01 | 8/9 | 6 (75) | 7 (87.5) | 6 (75) |
| S02 | 5/9 | 4 (80) | 4 (80) | 4 (80) |
| M01 | 3/10 | 3 (100) | 3 (100) | 3 (100) |
| M02 | 2/10 | 2 (100) | 2 (100) | 2 (100) |
| M03 | 2/13 | 2 (100) | 2 (100) | 2 (100) |
| M04 | 6/23 | 6 (100) | 6 (100) | 6 (100) |
| M05 | 2/16 | 2 (100) | 2 (100) | 2 (100) |
| M06 | 2/13 | 2 (100) | 2 (100) | 2 (100) |
| M07 | 2/33 | 2 (100) | 2 (100) | 2 (100) |
| M08 | 2/3 | 2 (100) | 2 (100) | 2 (100) |
| M09 | 2/3 | 2 (100) | 2 (100) | 2 (100) |
| M10 | 3/5 | 3 (100) | 3 (100) | 3 (100) |
| M11 | 2/5 | 2 (100) | 2 (100) | 2 (100) |
| H02 | 2/5 | 2 (100) | 2 (100) | 2 (100) |
| H03 | 3/5 | 3 (100) | 3 (100) | 3 (100) |
| Total | 33/164 |  |  |  |

.
